# IP_3_R-Grp75-VDAC1-MCU calcium regulation axis antagonists protect podocytes from apoptosis and decrease proteinuria in an Adriamycin nephropathy rat model

**DOI:** 10.1186/s12882-018-0940-3

**Published:** 2018-06-15

**Authors:** Han Xu, Na Guan, Ya-Li Ren, Qi-Jiao Wei, Ying-Hong Tao, Guo-Sheng Yang, Xiao-Ya Liu, Ding-Fang Bu, Ying Zhang, Sai-Nan Zhu

**Affiliations:** 1 Department of Pediatrics, Peking University First Hospital, Beijing, 100034 China; 2 Lab of Electron Microscope, Peking University First Hospital, Beijing, 100034 China; 3 Experimental Animal Center, Peking University First Hospital, Beijing, 100034 China; 4 Central Laboratory, Peking University First Hospital, Beijing, 100034 China; 5 Department of Biostatistics, Peking University First Hospital, Beijing, 100034 China

**Keywords:** Podocyte, Apoptosis, Endoplasmic reticulum mitochondria coupling, Mitochondria, Calcium

## Abstract

**Background:**

The mechanism of podocyte apoptosis is not fully understood. In addition, the role of the inositol 1,4,5-triphosphate receptor (IP_3_R)/glucose-regulated protein 75 (Grp75)/voltage-dependent anion channel 1 (VDAC1)/mitochondrial calcium uniporter (MCU) calcium regulation axis, which is located at sites of endoplasmic reticulum (ER) mitochondria coupling, in the mechanism of podocyte apoptosis is unclear. This study aimed to understand the roles of this axis in podocyte apoptosis and explore potential targets for podocyte protection.

**Methods:**

The expression of IP_3_R, Grp75, VDAC1, and MCU and mitochondrial Ca^2+^ were analyzed during Adriamycin- or angiotensin II-induced apoptosis in cultured mouse podocytes. The interaction between IP_3_R, Grp75, and VDAC1 was investigated using co-immunoprecipitation experiments. The effects of IP_3_R, Grp75, and MCU agonists and antagonists on mitochondrial Ca^2+^ and apoptosis were investigated in cultured podocytes. The podocyte-protective effects of an MCU inhibitor were further investigated in rats with Adriamycin-induced nephropathy.

**Results:**

Increased expression of IP_3_R, Grp75, VDAC1 and MCU, enhanced interaction among the IP_3_R-Grp75-VDAC1 complex, mitochondrial Ca^2+^ overload, and increased active caspase-3 levels were confirmed during Adriamycin- or angiotensin II-induced mouse podocyte apoptosis. Agonists of this axis facilitated mitochondrial Ca^2+^ overload and podocyte apoptosis, whereas specific antagonists against IP_3_R, Grp75, or MCU prevented mitochondrial Ca^2+^ overload and podocyte apoptosis. A specific MCU inhibitor prevented Adriamycin-induced proteinuria and podocyte foot process effacement in rats.

**Conclusions:**

This study identified a novel pathway in which the IP_3_R-Grp75-VDAC1-MCU calcium regulation axis mediated podocyte apoptosis by facilitating mitochondrial Ca^2+^ overload. Antagonists that inhibit Ca^2+^ transfer from ER to mitochondria protected mouse podocytes from apoptosis. An MCU inhibitor protected podocytes and decreased proteinuria in rats with Adriamycin-induced nephropathy. Therefore, antagonists to this pathway have promise as novel podocyte-protective drugs.

## Background

Treatment-resistant nephrotic syndrome is a challenging clinical problem. Several patients with nephrotic syndrome are resistant to current therapies such as corticosteroid, calcineurin inhibitors, and mycophenolic acid, and they are at a higher risk of developing end stage renal disease [1]. Among patients with treatment-resistant nephrotic syndrome, focal segmental glomerulosclerosis is the most common pathological change. Podocyte apoptosis play a key role in the development of focal segmental glomerulosclerosis. However, the mechanism underlying podocyte apoptosis has not been fully clarified. Different mechanisms could mediate podocyte apoptosis, such as cytosolic Ca^2+^ overload [2], mitochondrial dysfunction, endoplasmic reticulum (ER) stress, and oxidative stress [3].

Ca^2+^ functions as a second messenger and plays a central role mediating apoptosis. Ca^2+^ homeostasis is mainly regulated by membrane Ca^2+^ channels and intracellular Ca^2+^ stores; ER, mitochondria, lysosomes, endosomes and Golgi apparatus are the main Ca^2+^ stores. Among the Ca^2+^ stores, the transfer of Ca^2+^ from the ER to mitochondria via close contacts between the two organelles named, ER-mitochondria coupling, plays a critical role in maintaining intracellular Ca^2+^ homeostasis. The coupling occurs via mitochondria-associated membranes (MAMs), which account for ~ 20% of the mitochondrial surface. A single yeast cell contains around 100 couplings. Under normal physiological conditions, the cytosolic Ca^2+^ concentration is ~ 100 nM, the ER Ca^2+^ concentration is ~ 1000 μM, and the mitochondrial Ca^2+^ concentration is ~ 1000 nM [4]. Ca^2+^ transfer from the ER to the mitochondria is required for mitochondrial ATP production and maintaining cell survival, but excessive Ca^2+^ entrance into the mitochondria can lead to mitochondrial Ca^2+^ overload and trigger opening of the mitochondrial permeability transition pore, release of pro-apoptotic factors such as cytochrome c, caspase activation, and apoptosis. This pathway of mitochondrial Ca^2+^-dependent cell death is crucial in a plethora of cell types [4–8].

The molecular basis of Ca^2+^ transfer from the ER to mitochondria is the inositol 1,4,5 triphosphate receptor (IP_3_R)/glucose-regulated protein 75 (Grp75)/voltage dependent anion channel 1 (VDAC1) complex, which is located at sites of ER-mitochondrial coupling. IP_3_R is the Ca^2+^ release channel located at the ER membrane, VDAC1 is the channel located at the outer mitochondrial membrane, and Grp75 is a bridging protein that interacts physically with IP_3_R and VDAC1. The Ca^2+^ released from the ER forms Ca^2+^ hot spots between the ER and the mitochondria, enters the mitochondrial intermembrane space, and then finally enters the mitochondrial matrix via the mitochondrial calcium uniporter (MCU) [5]. Based on the route of Ca^2+^ transfer, we call IP_3_R-Grp75-VDAC1-MCU the intracellular calcium regulation axis.

Little is known about the roles of the IP_3_R-Grp75-VDAC1-MCU axis in the mechanism of podocyte apoptosis. In the current study, the roles of the IP_3_R-Grp75-VDAC1-MCU calcium regulation axis in podocyte apoptosis were investigated in cultured mouse podocytes exposed to Adriamycin (ADR) and angiotensin II (Ang II) in vitro. Further studies on proteinuria regulation and the protective effects of calcium regulation axis antagonists on podocytes were performed in an ADR nephropathy rat model.

## Methods

### Induction of mouse podocyte apoptosis in vitro

Mouse podocytes (Endlich mouse podocytes, a generous gift from Prof. Hong Hui Wang from Hunan University, China) were cultured as reported previously [9]. Briefly, the podocytes were cultured at 33°C in RPMI-1640 medium supplemented with 10% fetal bovine serum, 100 U/ml penicillin/streptomycin, and 10 U/ml recombinant mouse interferon-γ (IFN-γ) to induce proliferation for 7 days. Then, they were transferred to culture media lacking IFN-γ at 37°C to differentiate for 10–14 days. To induce apoptosis, the podocytes were treated with 0.5 μg/ml ADR (D1515, Sigma, Santa Clara, California, USA) or 1 μM Ang II (A9525, Sigma) for 24 h.

### Analysis of podocyte apoptosis by flow cytometry

Cells were washed twice with cold PBS and then resuspended in 1× binding buffer (556,547, BD Biosciences, Franklin Lake, New Jersey, USA) at a concentration of 1 × 10^6^ cells/ml. Then, a 100 μl cell suspension was transferred to a 5-ml culture tube. The podocytes were stained with 5 μl FITC-conjugated annexin V and 5 μl propidium iodide (PI, 556547, BD Biosciences) for 15 min at room temperature in the dark and analyzed by flow cytometry (Flow Cytometer, FACSCanto II, BD Biosciences) within 1 h to analyze apoptosis [10]. When doing flow cytometry, naked cells without staining, cells stained with FITC-annexin V only and cells stained with PI only were used for adjustment of the detecting parameters of flow cytometry in advance. The cells which were annexin high and PI low were counted as apoptotic cells. The apoptotic rate of podocytes among different groups were compared.

### Analysis of mitochondrial Ca^2+^-related apoptosis

Podocytes were cultured in 96 well plates at 1 × 10^5^ cells/well for 24 h, treated with different drugs or transfection and used for mitochondrial Ca^2+^ detection. Mitochondrial Ca^2+^ was labeled with Rhod-2 AM (Invitrogen, Karlsruhe, Germany) [11]. The working solution was dispensed from a 1 mM DMSO stock and diluted to 5 μM in PBS. Podocytes were incubated with 5 μM Rhod-2 AM at 4°C for 30 min, washed, and detected using Synergy 4 Multi-Detection Microplate Reader (Synergy 4, BioTek, Vermont, USA). Rhod-2 AM was detected using excitation at 552 nm and emission at 581 nm. Before labeling of Rhod-2 AM, the background fluorescence intensity of cells in each well was measured as F0. After labeling of Rhod-2 AM, the fluorescence intensity of cells in each well was measured as F1. The value of F1-F0 was used to reflect mitochondrial Ca^2+^ level of cells in each well.

Western blotting was used to detect active caspase-3 [10]. Rabbit polyclonal anti-active caspase-3 (ab2302, Abcam, Cambridge, UK) and mouse monoclonal anti-GAPDH (RM2002, Beijing Ray Antibody Biotech) were used. Total cellular proteins were extracted using non-denatured RIPA lysis buffer containing protease inhibitor and then quantified using a Pierce™ BCA Protein Assay Kit (ThermoFisher Scientific, Waltham, Massachusetts, USA). After SDS-PAGE electrophoresis on 6–15% gels, the proteins were transferred to nitrocellulose membranes using the Mini Trans Blot Cell (Bio-Rad). The membranes were blocked with 5% BSA/PBS-T for 60 min and then incubated overnight at 4°C with primary antibodies. After three washes with PBST, the membranes were incubated with horseradish peroxidase-conjugated secondary antibodies (Applygen Technologies Inc., Beijing, China) for 1 h at room temperature and the signal was developed using an ECL chemiluminescence detection kit (Millipore, Massachusetts, USA). The ratio of active caspase-3/GAPDH was semi-quantitated using ImageJ software.

### Western blot analysis of expression of IP_3_R, Grp75, VDAC1, and MCU

For western blot analysis, rabbit polyclonal anti-IP_3_R (ab5804, Abcam), rabbit monoclonal anti-Grp75 (D13H4, #3593, Cell Signaling Technology), mouse monoclonal anti-VDAC1 (ab14734, Abcam), rabbit monoclonal anti-MCU (D2Z3B, #14997, Cell Signaling Technology), and mouse monoclonal anti-GAPDH antibodies were used. Western blotting and semi-quantitation were performed as the method described above.

### Co-immunoprecipitation (co-IP) analysis of the IP_3_R-Grp75-VDAC1 complex

Podocytes lysates (generating ~ 1.5 mg total protein) were collected using ice-cold IP lysis buffer (26,149, ThermoFisher Scientific**)**. The lysates were transferred to a micro centrifuge tube and centrifuged at ~ 13,000×*g* for 10 min to pellet the cell debris. Then, the supernatant was transferred to a new tube for protein concentration determination and further analysis. Co-IP was performed using a Thermo Scientific Pierce Co-IP Kit **(**26,149, ThermoFisher Scientific) according to the manufacturer’s protocols. Anti-Grp75 antibody was used as the bait antibody to capture mitochondria-ER coupling proteins. Rabbit monoclonal anti-Grp75 antibody (D13H4, #3593, Cell Signaling Technology) was first immobilized using AminoLink Plus Coupling Resin **(**26,149, ThermoFisher Scientific). Then, the resin was washed and incubated with lysate overnight. After incubation, the resin was washed again and proteins were eluted using Elution Buffer **(**26,149, ThermoFisher Scientific). Normal rabbit IgG without antigenicity provided with the kit was used as a negative control to detect nonspecific binding. The control was treated in the same way as the Co-IP samples, including incubation with the Grp75 antibody. After Co-IP, the proteins pulled down by anti-Grp75 antibodies were analyzed by western blotting [12, 13]. Lysates from both Ctl and ADR- or Ang-II treated podocytes without immunoprecipitation were used as a positive control (input).

### IP_3_R-Grp75-VDAC1-MCU axis agonists

D-myo-inositol 1,4,5-triphosphate tripotassium salt (IP_3_, 74,148, Sigma) was used at a concentration of 10 μM diluted in ultra-pure water to stimulate IP_3_R in cultured mouse podocytes for 24 h. Spermine (S3256, Sigma) was used at a concentration of 20 μM, diluted in ultra-pure water, to stimulate MCU in cultured mouse podocytes for 2 h.

### IP_3_R-Grp75-VDAC1-MCU axis antagonists

The IP_3_R inhibitor Xestospongin C (XeC, ×2628, 10 μM, Sigma) [14] and the MCU inhibitor Ru360 (557,440, 10 μM, Merck, Kenilworth, New Jersey, USA) [15] were used to block ER calcium release and mitochondrial Ca^2+^ uptake, respectively. Podocytes were pre-treated with the above inhibitors for 60 min before treatment with ADR or Ang II, respectively. Specific siRNA targeting the bridging protein Grp75 and a non-targeted negative control siRNA were synthesized by Invitrogen. Podocytes were plated in six-well plates and treated with 100 pmol/well siRNA duplexes using 10 μl RNAiMAX reagent (Invitrogen) according to the manufacturer’s protocol. After 8–12 h, the media were changed according to the status of cell growth at 40–50% confluence. The podocytes were collected for further experiments 24 h after transfection.

### ADR nephropathy rat model and MCU inhibitor treatment

All protocols were approved by the Institutional Animal Care and Use Committee of Peking University First Hospital (Number: 11400700229305). Ruthenium red (RR, R2751, Sigma) was used as a specific inhibitor of MCU. Thirty-two male Sprague Dawley rats weighing 80–100 g were randomly divided into four groups: normal saline control (Ctl, *n* = 6), RR control (RR, *n* = 6), ADR group (ADR, *n* = 10), and ADR plus RR (ADR + RR, *n* = 10). The rats were fed a standard diet, and water was given ad libitum; they were maintained using alternating 12-h cycles of light and dark. After acclimatization for 48 h, the rats in ADR and ADR + RR group received a tail vein injection of 0.8 mg/100 g bodyweight ADR in sterile water (the 0 time point). Immediately after ADR injection, rats in the ADR + RR group were administered RR at a dose of 2.5 mg/kg/d by intraperitoneal injection for 14 days. The rats in Ctl and RR groups received normal saline and RR injections for 14 days, respectively. All rats were sacrificed at the 6-week time point and the kidneys were harvested. Twenty-four hours urine was collected from each rat at 0, 2 weeks, 4 weeks, and 6 weeks using metabolic cages.

### Effects of RR on proteinuria and podocyte in ADR nephropathy

Urinary proteins were analyzed using the Pyrogallol red-molyb-date dye-binding method and a Hitachi-7150 Automatic biochemical analyzer (Hitachi, Tokyo, Japan). Ultrathin sections of renal cortex were made for electron microscopy using a method reported previously [10]. Averaged 24 electron microscopic photographs of glomeruli were taken randomly from each rat. Podocyte foot process width was analyzed as reported previously [16] using Olympus Scandium SEM imaging software. Total glomerular basement membrane length was measured. Total number of foot processes in each glomerular basement membrane was counted. Total glomerular basement membrane length divided by number of foot process was used as mean foot process width.

### Statistical analysis

SPSS20.0 statistical analysis software was used for all analyses. All data are presented as means ± SD. Unpaired Student’s *t*-tests were used to compare differences between two groups. One-way ANOVA was used to compare differences among more than two groups. *P* < 0.05 was used to define statistical significance.

## Results

### Enhanced expression and interaction of the IP_3_R-Grp75-VDAC1-MCU calcium regulation axis mediated podocyte apoptosis by facilitating mitochondrial Ca^2+^ overload

**Fig. 1:**
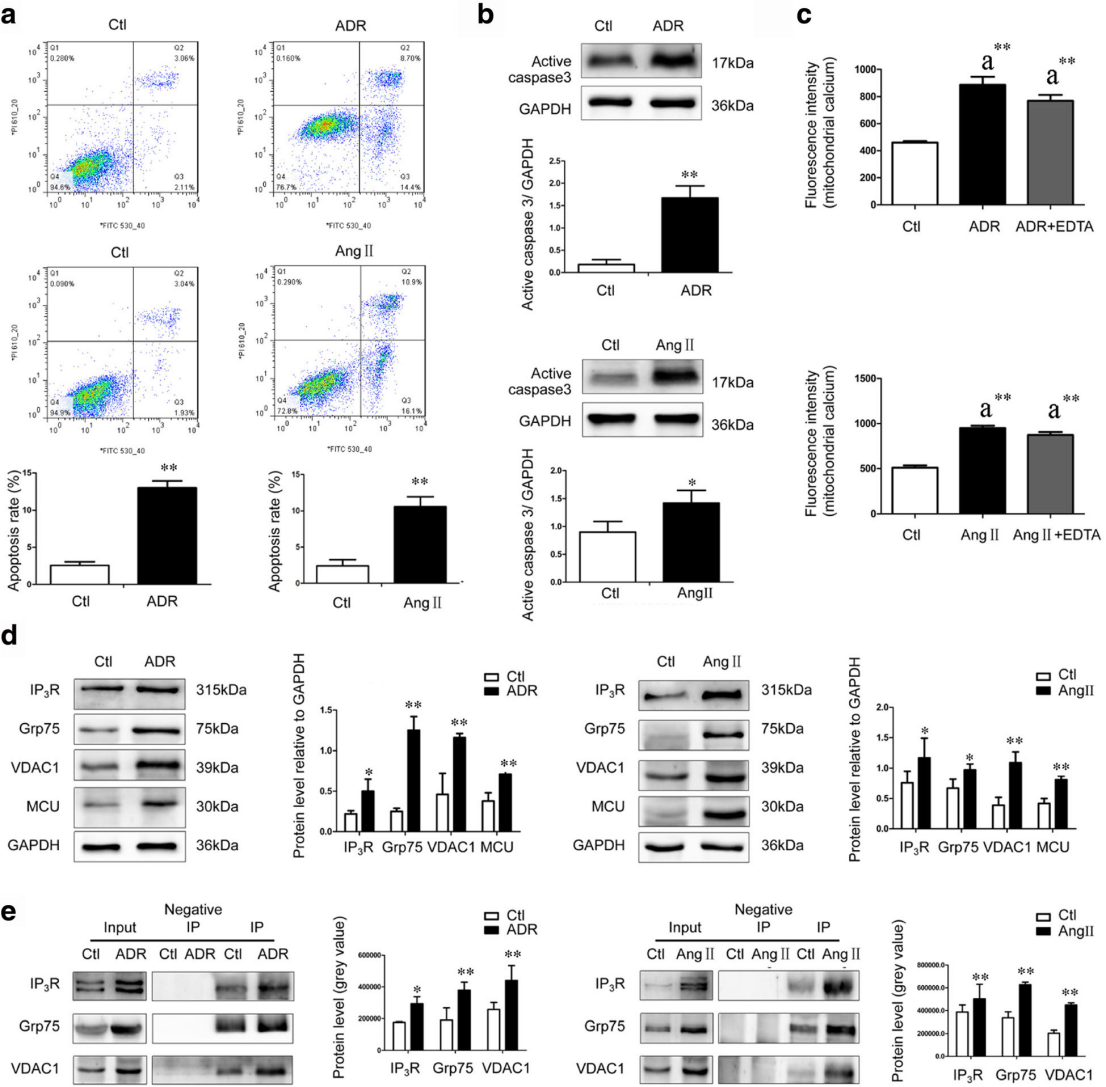
IP_3_R-Grp75-VDAC1-MCU axis, mitochondrial Ca^2+^, and apoptosis in podocytes treated with ADR and Ang II. Ctl, control; ADR, Adriamycin; Ang II, angiotensin II; IP_3_R, inositol 1,4,5-triphosphate receptor; Grp75, glucose-regulated protein 75; VDAC1, voltage dependent anion channel 1; MCU, mitochondrial calcium uniporter; a, compared with the Ctl group; **, *P* < 0.01; *, *P* < 0.05. Compared with the Ctl podocytes, **a**) The cells in Q3 which were annexin high and PI low were counted as apoptotic cells. Significantly increased apoptosis rate was found in ADR- (*n* = 3) and Ang II-treated podocytes (*n* = 3), **b**) Significantly increased levels of active caspase-3 was found in podocytes treated with ADR (*n* = 4) and Ang II (*n* = 4), **c**) Mitochondrial Ca^2+^ levels were increased in podocytes treated with ADR (*n* = 12) and Ang II (*n* = 12), **d**) Significantly increased expression of IP_3_R, Grp75, VDAC1, and MCU were found in podocytes treated with ADR (*n* = 6) and Ang II (*n* = 6). **e** Co-IP were performed to analyze the IP_3_R-Grp75-VDAC1 interaction. Normal rabbit IgG without antigenicity was used as a negative control. Lysates from both Ctl and ADR- or Ang-II treated podocytes without immunoprecipitation were used as a positive control (input). The proteins pulled down by anti-Grp75 antibodies was analyzed by western blotting. Compared with the Ctl podocytes, there was a significant increase in the amount of IP_3_R, Grp75, and VDAC1 in pulled down samples from ADR- (*n* = 3) or Ang II-treated podocytes (*n* = 3)

Compared with normal Ctl podocytes, the apoptosis rate revealed by flow cytometry in mouse podocytes treated with ADR (13.02% ± 0.93% vs. 2.56% ± 0.49%, *P* = 0.000, *n* = 3) or Ang II (10.58% ± 1.38% vs. 2.40% ± 0.85%, *P* = 0.001, *n* = 3) increased significantly (Fig. 1a), active caspase-3 level revealed by western blotting in mouse podocytes treated with ADR (*P* = 0.006, *n* = 4) or Ang II (*P* = 0.021, *n* = 4) increased significantly (Fig. 1b).

Compared with the Ctl podocytes, mitochondrial Ca^2+^ revealed by Rhod-2 AM fluorescence intensity in mouse podocytes treated with ADR (*P* = 0.000, *n* = 12) or Ang II (*P* = 0.000, *n* = 12) increased significantly (Fig. 1c). To exclude any effect of podocyte membrane Ca^2+^ channels on increase of mitochondrial Ca^2+^, EDTA (Sigma) was used to chelate extracellular Ca^2+^. However, compared with the podocytes treated with ADR only (*P* = 0.124, *n* = 12) or Ang II only (*P* = 0.083, *n* = 12), the increase in mitochondrial Ca^2+^ induced by ADR or Ang II was not prevented by additional treatment with EDTA (Fig. 1c).

Compared with the Ctl podocytes, increased expression of IP_3_R (*P* = 0.040, *n* = 6), Grp75 (*P* = 0.001, *n* = 6), VDAC1 (*P* = 0.009, *n* = 6) and MCU (*P* = 0.008, *n* = 6) was observed during ADR-induced podocyte apoptosis (Fig. 1d), increased expression of IP_3_R (*P* = 0.041, *n* = 6), Grp75 (*P* = 0.045, *n* = 6), VDAC1 (*P* = 0.007, *n* = 6) and MCU (*P* = 0.002, *n* = 6) was also observed during Ang II-induced podocyte apoptosis (Fig. 1d).

In addition, Co-IP experiments revealed that the interaction among the IP_3_R-Grp75-VDAC1 complex was increased during ADR- and Ang II-induced podocyte apoptosis. Compared with the Ctl podocytes, the amount of IP_3_R (*P* = 0.010, *n* = 3), VDAC1 (*P* = 0.039, *n* = 3), and Grp75 (*P* = 0.024, *n* = 3) pulled down by anti-Grp75 antibodies was increased significantly during ADR-induced podocyte apoptosis (Fig. 1e), the amount of IP_3_R (*P* = 0.048, *n* = 3), VDAC1 (*P* = 0.000, *n* = 3), and Grp75 (*P* = 0.001, *n* = 3) pulled down by anti-Grp75 antibodies was also increased significantly during Ang II-induced podocyte apoptosis (Fig. 1e).

### IP_3_R and MCU agonists caused mitochondrial Ca^2+^ overload and apoptosis in mouse podocytes

**Fig. 2:**
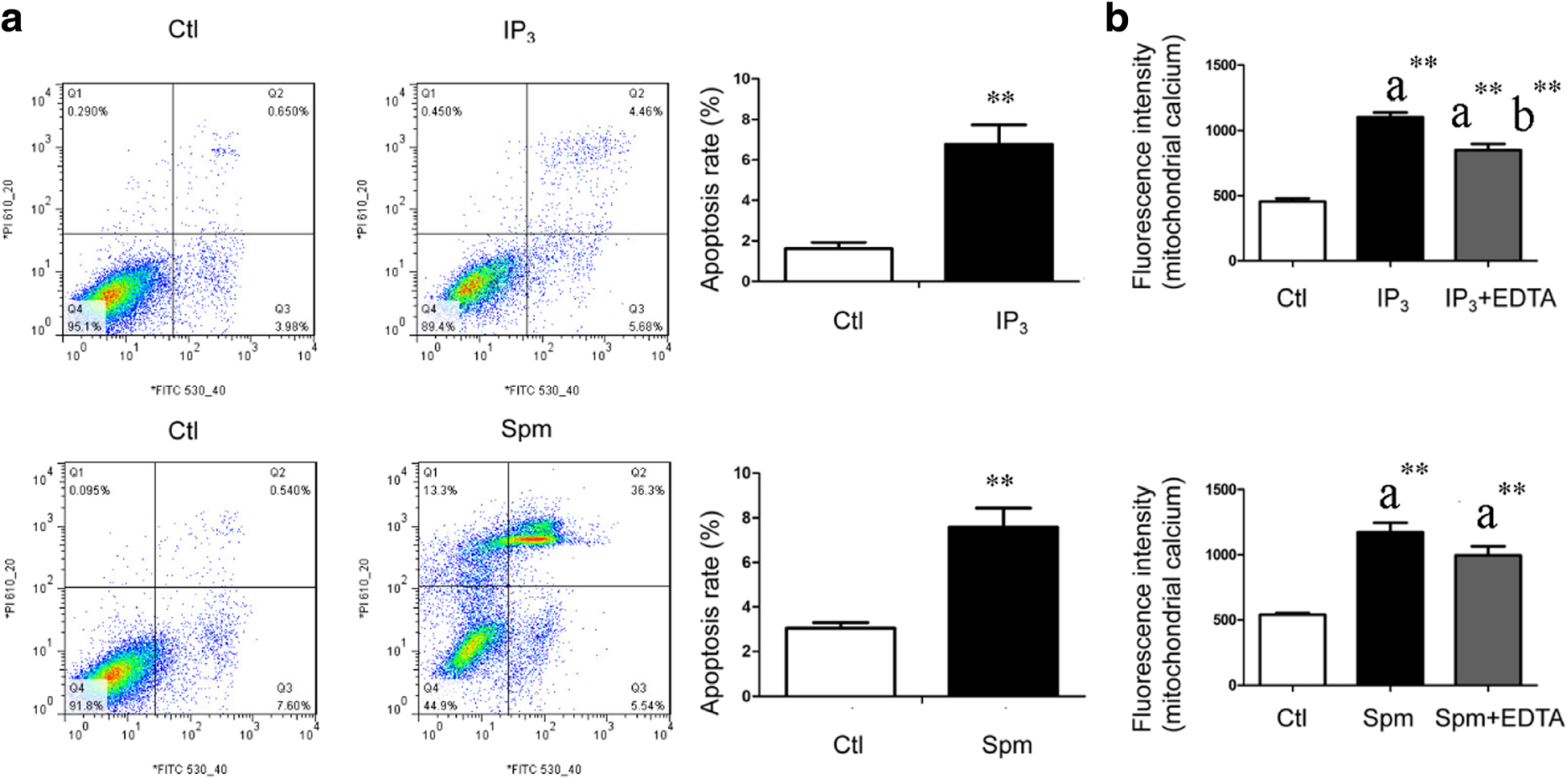
IP_3_ and MCU agonists induced podocyte mitochondrial Ca^2+^ overload and apoptosis in vitro. Ctl, control; IP_3_: D-myo-inositol 1, 4, 5-triphosphate tripotassium salt; Spm: Spermine; a, compared with the Ctl group; b, compared with the IP_3_ group; **, *P* < 0.01; *, *P* < 0.05. **a** The cells in Q3 which were annexin high and PI low were counted as apoptotic cells. Compared with the Ctl podocytes, IP_3_ (*n* = 9) and Spm (*n* = 3) all induced significant increase of the podocyte apoptosis rate. **b** Compared with the Ctl podocytes, IP_3_ and Spm all induced significant increase of mitochondrial Ca^2+^ in mouse podocytes (*n* = 12). The increase of mitochondrial Ca^2+^ induced by IP_3_ was partially prevented by EDTA

Compared with the Ctl podocytes, the IP_3_R agonist IP_3_ induced a significant increase of the podocyte apoptosis rate compared with the Ctl podocytes (6.78% ± 0.96% vs. 1.61% ± 0.32%, *P* = 0.000, *n* = 9, Fig. 2a). Also, IP_3_ induced a significant increase in mitochondrial Ca^2+^ levels (*P* = 0.000, *n* = 12) in mouse podocytes, which was partially prevented by treatment with the Ca^2+^ chelator EDTA (*P* = 0.001, *n* = 12, Fig. 2b).

Compared with the Ctl podocytes, apoptosis rate in MCU agonist Spermine treated podocytes increased significantly (7.60% ± 0.85% vs. 3.05% ± 0.25%, *P* = 0.001, *n* = 3, Fig. 2a). The Spermine also induced a significant increase in mitochondrial Ca^2+^ levels (*P* = 0.000, *n* = 12), which was not prevented by EDTA (*P* = 0.093, *n* = 12, Fig. 2b).

### IP_3_R inhibitor protected podocytes from apoptosis induced by ADR or Ang II

**Fig. 3:**
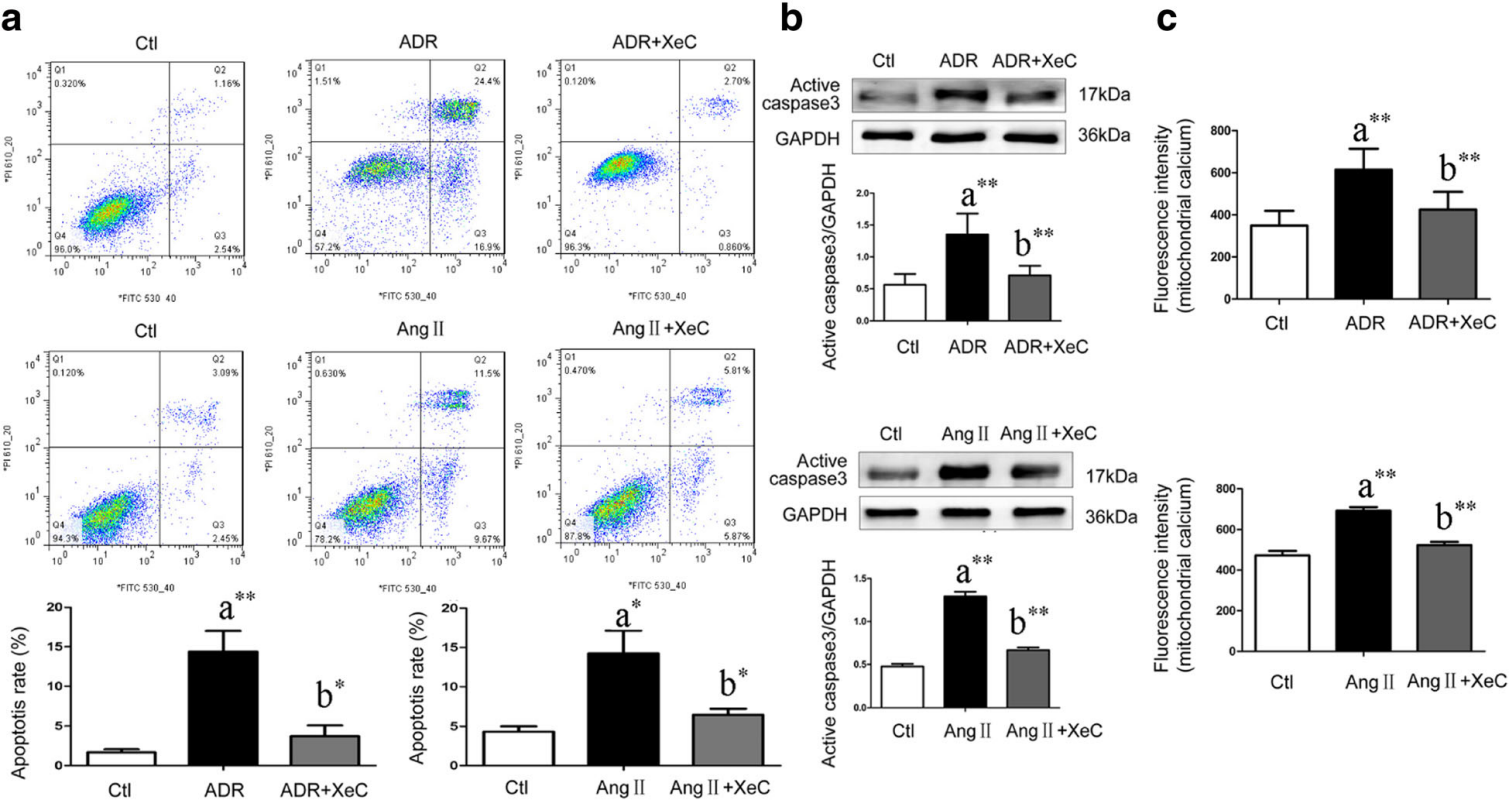
IP_3_R antagonist prevented podocytes from ADR- and Ang II-induced apoptosis in vitro. Ctl, control; ADR, Adriamycin; XeC, Xestospongin C (IP_3_R inhibitor); a, compared with Ctl group; b, compared with the ADR or Ang II group; *, *P* < 0.05; **, *P* < 0.01. **a** The cells in Q3 which were annexin high and PI low were counted as apoptotic cells. Compared with ADR or Ang II -treated podocytes, XeC decreased the podocyte apoptosis rate significantly (*n* = 3). **b** Compared with ADR or Ang II -treated podocytes, XeC decreased active caspase-3 in podocytes significantly. **c** Compared with ADR or Ang II -treated podocytes, XeC decreased mitochondrial Ca^2+^ levels in podocytes significantly

Compared with podocytes treated with ADR only, additional treatment with XeC decreased the podocyte apoptosis rate (3.73% ± 1.36% vs. 14.40% ± 2.60%, *P* = 0.022, *n* = 3, Fig. 3a), active caspase-3 (*P* = 0.004, *n* = 7, Fig. 3b), and mitochondrial Ca^2+^ levels (*P* = 0.005, *n* = 6, Fig. 3c) in mouse podocytes significantly.

During Ang II-induced podocyte apoptosis, treatment with XeC also prevented the increase in apoptosis rate (6.46% ± 0.78% vs. 14.26% ± 2.89%, *P* = 0.036, *n* = 3, Fig. 3a), active caspase-3 (*P* = 0.000, *n* = 4, Fig. 3b), and increased mitochondrial Ca^2+^ levels (*P* = 0.000, *n* = 6, Fig. 3c).

### MCU inhibitor protected podocytes from apoptosis induced by ADR or Ang II

**Fig. 4:**
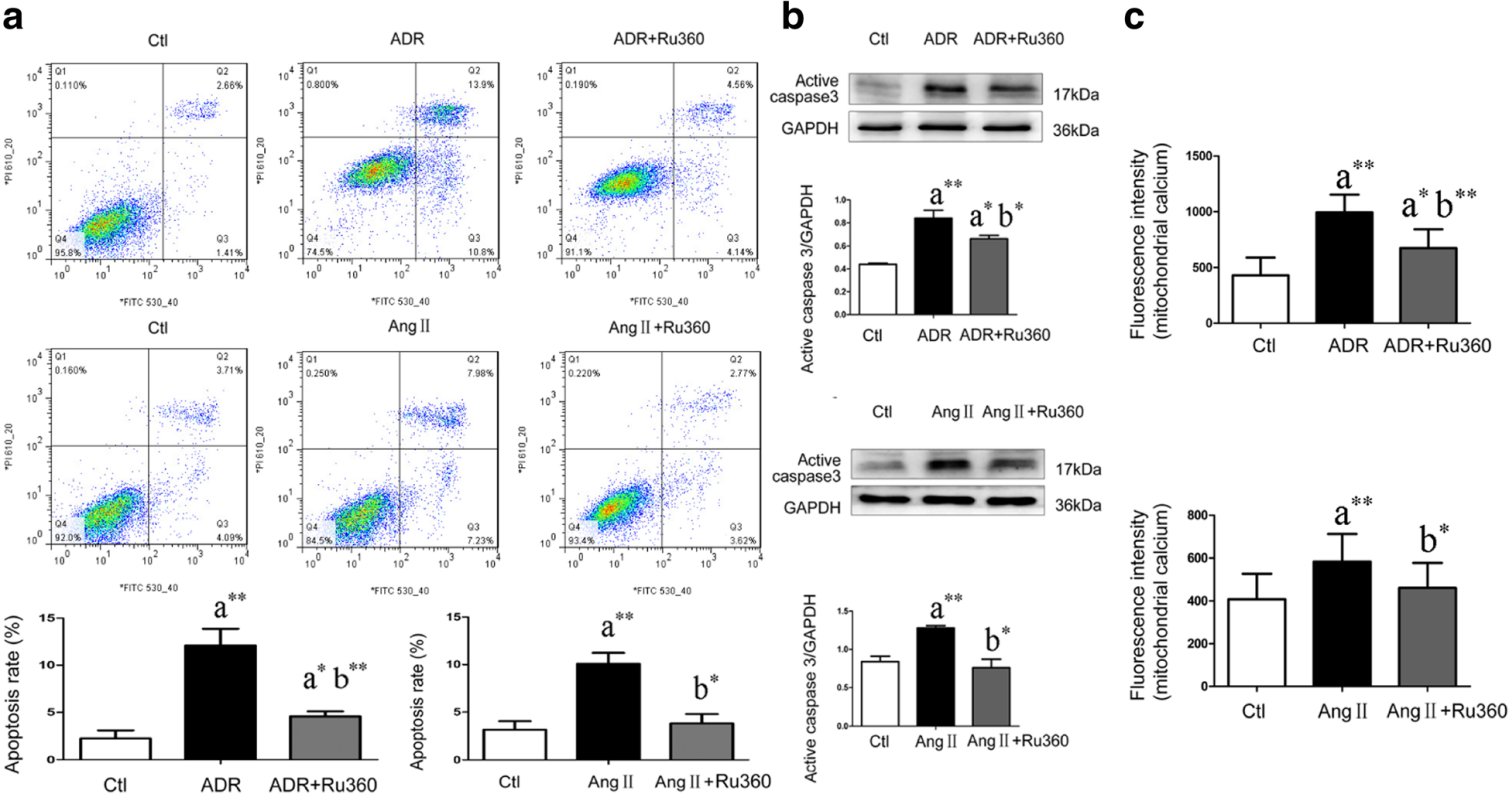
MCU antagonist prevented podocytes from ADR- and Ang II-induced apoptosis in vitro. Ctl, control; ADR, Adriamycin; Ang II, angiotensin II; Ru360, MCU inhibitor; a, compared with Ctl group; b, compared with the ADR or Ang II group; *, *P* < 0.05; **, *P* < 0.01. **a** The cells in Q3 which were annexin high and PI low were counted as apoptotic cells. Compared with ADR or Ang II -treated podocytes, Ru360 decreased the podocyte apoptosis rate significantly (*n* = 3). **b** Compared with ADR or Ang II -treated podocytes, Ru360 decreased active caspase-3 in mouse podocytes significantly (*n* = 4). **c** Compared with ADR or Ang II -treated podocytes, Ru360 decreased mitochondrial Ca^2+^ levels in mouse podocytes significantly (*n* = 10)

Treatment with Ru360 prevented the ADR-induced increased podocyte apoptosis rate (4.57% ± 0.52% vs. 12.10% ± 1.77%, *P* = 0.002, *n* = 3, Fig. 4a), increase in active caspase-3 (*P* = 0.039, *n* = 4, Fig. 4b), and increased mitochondrial Ca^2+^ levels (*P* = 0.001, *n* = 10, Fig. 4c).

During Ang II-induced podocyte apoptosis, additional treatment with Ru360 also prevented increased apoptosis rate (3.84% ± 0.96% vs. 10.08% ± 1.15%, *P* = 0.014, *n* = 3, Fig. 4a), the increase in active caspase-3 (*P* = 0.003, *n* = 4, Fig. 4b), and increased mitochondrial Ca^2+^ levels (*P* = 0.031, *n* = 10, Fig. 4c).

### Knocking down Grp75 protected podocytes from apoptosis induced by ADR or Ang II

**Fig. 5:**
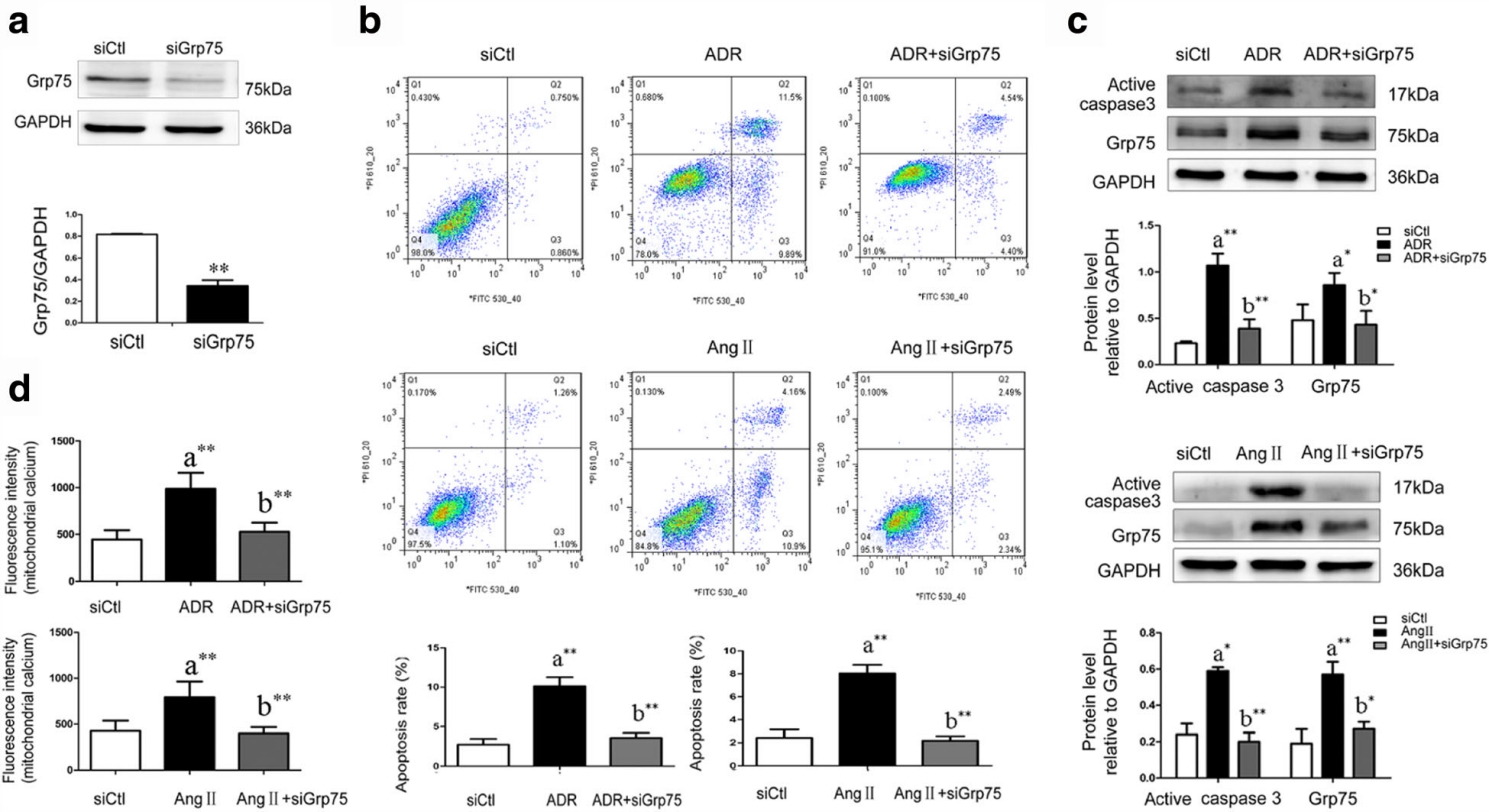
Knocking down Grp75 protected podocytes from ADR- and Ang II-induced apoptosis in vitro. Ctl, control; Ang II, angiotensin II; Grp75, glucose-regulated protein 75; siCtl, non-targeted negative control siRNA; siGrp75, siRNA to knock down Grp75; a, compared with the Ctl group; b, compared with the ADR or Ang II group; *, *P* < 0.05; **, *P* < 0.01. **a** Knocking down Grp75 decreased Grp75 compared with non-targeted negative control siRNA group**; b**) The cells in Q3 which were annexin high and PI low were counted as apoptotic cells. Compared with ADR or Ang II -treated podocytes, knock down of Grp75 decreased the podocyte apoptosis rate significantly. **c** Compared with ADR or Ang II -treated podocytes, knock down of Grp75 decreased active caspase-3 significantly. **d** Compared with ADR or Ang II -treated podocytes, knock down of Grp75 decreased mitochondrial Ca^2+^ levels significantly

Knocking down Grp75 decreased Grp75 compared with non-targeted negative control siRNA group (0.34 ± 0.09 vs. 0.82 ± 0.01, *P* = 0.000, *n* = 3, Fig. 5a), prevented the ADR-induced increased apoptosis rate (3.58% ± 0.62% vs. 10.13% ± 1.12%, *P* = 0.001, *n* = 7, Fig. 5b), increase in active caspase-3 (*P* = 0.003, *n* = 3, Fig. 5c), and increased mitochondrial Ca^2+^ levels (*P* = 0.000, *n* = 10, Fig. 5d).

During Ang II-induced podocyte apoptosis, knocking down Grp75 also prevented the increased apoptosis rate (2.18% ± 0.38% vs. 8.04% ± 0.74%, *P* = 0.000, *n* = 6, Fig. 5b), increase in active caspase-3 (*P* = 0.000, *n* = 3, Fig. 5c), and increased mitochondrial Ca^2+^ levels (*P* = 0.000, *n* = 8, Fig. 5d).

### RR prevented proteinuria and podocyte foot process effacement in ADR nephropathy rats

**Fig. 6:**
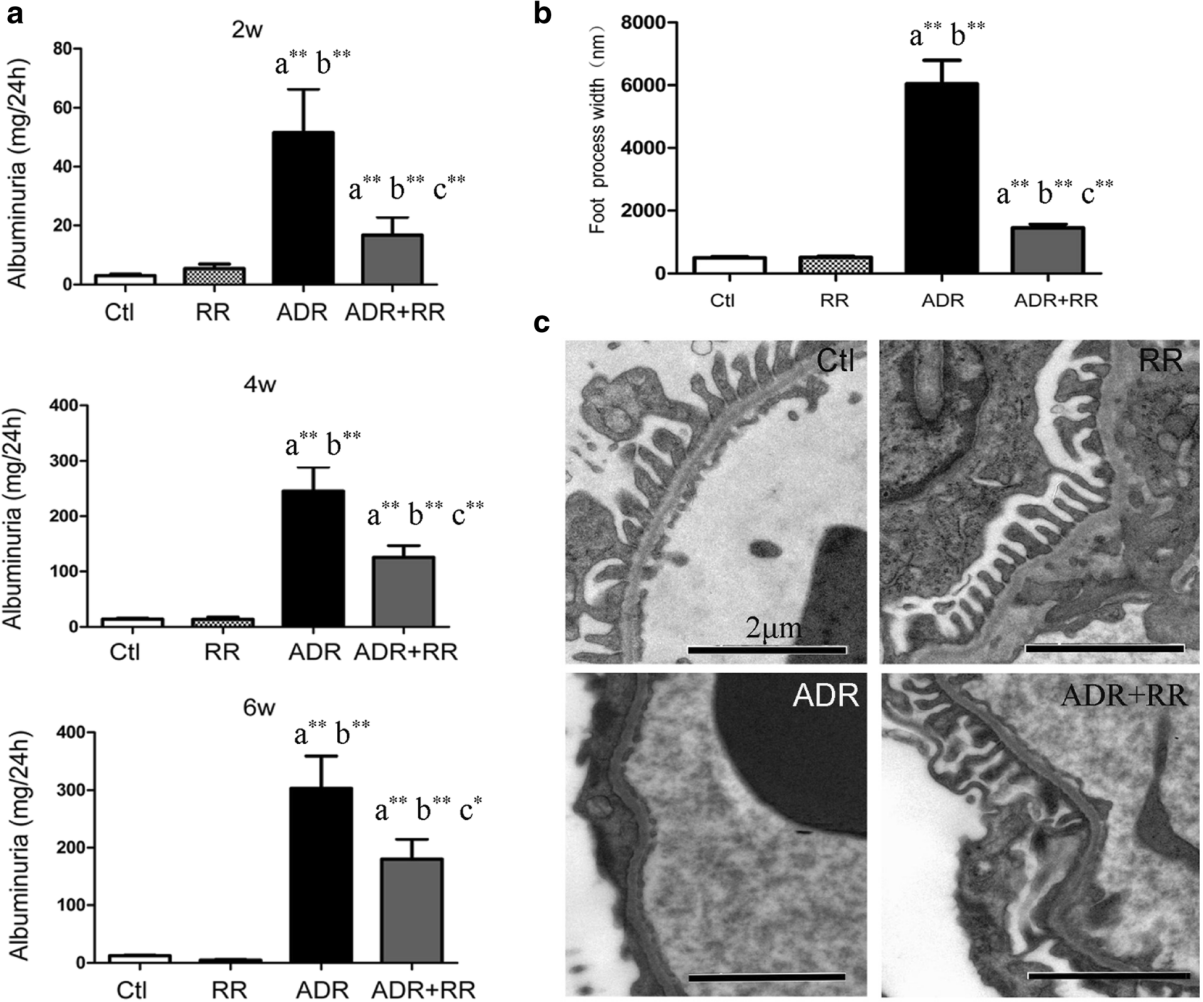
The protective effects of ruthenium red in an ADR nephropathy rat model. W, week; Ctl, normal saline group; RR, ruthenium red group; ADR, Adriamycin group; ADR + RR, Adriamycin plus RR group. a, compared with the Ctl group; b, compared with the RR group; c, compared with the ADR group. *, *P* < 0.05; **, *P* < 0.01. **a** Compared with the Ctl (*n* = 6) or RR (*n* = 6) groups, 24 h urinary protein levels increased significantly in the ADR group (*n* = 10) at 2, 4, and 6-weeks. Compared with the ADR group, 24 h urinary protein levels were decreased significantly in rats in the ADR + RR (*n* = 6) group at 2, 4, and 6 weeks. **b and c** Compared with the Ctl (*n* = 6) or RR (*n* = 6) groups, significantly increased podocyte foot process width was observed in rats in the ADR group (*n* = 6). Compared with the ADR group, podocyte foot process width was decreased significantly in rats in the ADR + RR group (*n* = 6)

In the ADR + RR group, four rats died of unknown reasons before the end of the experiment and six rats survived. Compared with the corresponding Ctl group (*n* = 6), there were increased levels of urinary proteins in rats in the ADR group (*n* = 10) at 2 (*P* = 0.000), 4 (*P* = 0.000), and 6-weeks (*P* = 0.000), but RR alone (*n* = 6) did not induce proteinuria. Compared with the ADR group, urinary protein levels in rats in the ADR + RR group (*n* = 6) were decreased significantly at 2 (*P* = 0.000), 4 (*P* = 0.000), and 6 (*P* = 0.017) weeks (Fig. 6a, Table 1).

**Table 1:** Comparison of 24-h urinary protein (mg) levels among different groups of rats

Group	2 weeks	4 weeks	6 weeks	*n*
Ctl	3.04 ± 0.49	14.09 ± 1.60	12.52 ± 0.90	6
RR	5.45 ± 1.54	13.83 ± 3.88	4.93 ± 1.00	6
ADR	51.60 ± 14.77^a**b**^	245.35 ± 43.81^a**b**^	303.00 ± 56.60^a**b**^	10
ADR + RR	16.69 ± 6.12 ^a**b**c**^	125.39 ± 21.49 ^a**b**c**^	180.18 ± 34.72 ^a**b**c*^	6

*Ctl* normal saline control group, *RR* ruthenium red group, *ADR* adriamycin group, *ADR + RR* adriamycin plus RR group. a, compared with the Ctl group; b, compared with the RR group; c, compared with the ADR group. *, *P* < 0.05; **, *P* < 0.01

All the rats in the Ctl, RR and ADR + RR group were included in electron microscopic analysis. Considering that 6 rats are enough for quantitation analysis, 6 rats from the ADR group were randomly used for electron microscopic analysis. Compared with the Ctl (504.63 ± 30.70 nm, *n* = 6) and RR (517.00 ± 31.53 nm, *n* = 6) groups, podocyte foot process width was increased significantly in rats in the ADR group (6035.15 ± 751.80 nm, *P* = 0.000, *n* = 6). Compared with the ADR group, podocyte foot process width was decreased significantly in rats in the ADR + RR group (1452.68 ± 115.36 nm, *P* = 0.000, *n* = 6, Fig. 6b, c).

## Discussion

Podocytes are core target cells for proteinuria control [1]. The mechanism underlying podocyte apoptosis has been investigated in different pathways [3]. Previous studies revealed that disturbance of cytosolic Ca^2+^ homeostasis, which is regulated by membrane Ca^2+^ channels, such as TRPC6 and TRPC5, is a key mediator of podocyte apoptosis or injury [2]. Recently, mounting evidence has demonstrated that dysregulation of Ca^2+^ transfer from the ER to mitochondria by the IP_3_R-Grp75-VDAC1-MCU axis, located at sites of ER-mitochondria coupling, will lead to mitochondrial Ca^2+^ overload, opening of mitochondrial permeability transition pore, the release of pro-apoptotic factors such as cytochrome c, caspase activation, and apoptosis in many different cells and diseases including neurodegeneration, metabolic diseases, and cancer [4–8]. However, it is unclear whether this pathway also mediates podocyte apoptosis.

For the first time, this study investigated the roles of the IP_3_R-Grp75-VDAC1-MCU calcium regulation axis during podocyte apoptosis. First, we explored whether this calcium regulation axis affects mitochondrial Ca^2+^ and apoptosis in podocytes by using IP_3_ to specifically stimulate Ca^2+^ release from IP_3_R located at the ER membrane and Spermine to specifically activate the mitochondria calcium uniporter. As expected, our results revealed that both agonists significantly increased mitochondrial Ca^2+^ levels and apoptosis rate in mouse podocytes. Second, to assess whether the IP_3_R-Grp75-VDAC1-MCU calcium regulation axis plays a role in podocyte apoptosis, we used the common apoptosis-inducing drugs ADR and Ang II. Western blotting identified significantly increased levels of IP_3_R, Grp75, VDAC1, and MCU during ADR- or Ang II-induced podocyte apoptosis. Next, Co-IP experiments revealed that the interaction among the IP_3_R, Grp75, and VDAC1 complex was enhanced, which suggests that these drugs may increase ER-mitochondria coupling during podocyte apoptosis. As previously described [5], ER-mitochondria coupling is the site through which Ca^2+^ transfer from the ER to mitochondria occurs; therefore, more coupling might lead to more Ca^2+^ transfer to the mitochondrial matrix. Indeed, significantly increased mitochondrial Ca^2+^ levels accompanied by increased active caspase-3 levels were found during ADR- and Ang II-induced mouse podocyte apoptosis. Together, these data revealed that the increased expression of IP_3_R, Grp75, VDAC1 and MCU, enhanced interaction of IP_3_R-Grp75-VDAC1, increased mitochondrial Ca^2+^ and active caspase-3 were observed during ADR- and Ang II-induced mouse podocyte apoptosis.

To demonstrate the possible causative effects of an enhanced IP_3_R-Grp75-VDAC1-MCU axis on mitochondrial Ca^2+^ overload and apoptosis in mouse podocytes, three different antagonists were used to inhibit different proteins in the axis. XeC is a specific IP_3_R inhibitor that decreases Ca^2+^ release from the ER. Si-Grp75 was used to knockdown Grp75 and hence decouple the IP_3_R-Grp75-VDAC1 axis [17]. RU360 is a specific MCU inhibitor that was used to block Ca^2+^ entry into the mitochondrial matrix. The current results clearly demonstrated that these three antagonists all prevented ADR- and Ang II-induced mitochondrial Ca^2+^ overload, increased active caspase-3 levels, and mouse podocyte apoptosis. These results confirmed that an enhanced IP_3_R-Grp75-VDAC1-MCU axis mediates mouse podocyte apoptosis by facilitating Ca^2+^ transfer from the ER to mitochondrial and mitochondrial Ca^2+^ overload. Therefore, antagonists of the IP_3_R-Grp75-VDAC1-MCU axis could prevent ADR- and Ang II-induced mitochondrial Ca^2+^ overload and apoptosis in mouse podocytes.

Little is known about the roles of this Ca^2+^ regulation axis in podocyte apoptosis. During palmitic acid-induced podocyte apoptosis, Yuan et al. [18] found that MCU was upregulated, which was accompanied by an increase in mitochondrial Ca^2+^ and cytochrome c levels. They also showed that inhibiting MCU prevented mitochondrial Ca^2+^ uptake and the mouse podocyte apoptosis induced by palmitic acid. To our knowledge, no studies have investigated the roles of Grp 75 and VDAC1 in podocyte apoptosis. Gong et al. [19] compared kidney proteins between diabetic and non-diabetic rats using method of proteomic analysis and found upregulation of VDAC1 in kidney proteins from diabetic rats. Further investigations into the mechanisms regulating the IP_3_R-Grp75-VDAC1-MCU calcium axis in podocytes are needed.

Finally, an ADR-induced nephropathy model was used to assess whether inhibiting the transfer of Ca^2+^ to mitochondrial matrix can protect against proteinuria. Since MCU is the final channel that regulates the entry of Ca^2+^ into the mitochondrial matrix, the MCU-specific inhibitor RR was used to treat ADR nephropathy rats. The dose of RR was selected based on a previous report in a subarachnoid hemorrhage rat model [20]. Interestingly, significantly decreased proteinuria accompanied by significantly improved podocyte foot process effacement was observed in the rats treated with ADR plus RR.

## Conclusions

This study identified a novel pathway mediating podocyte apoptosis and proteinuria involving Ca^2+^ transfer from the ER to mitochondria via the IP_3_R-Grp75-VDAC1-MCU axis located at sites of ER- mitochondria coupling. This study also revealed that increased expression of the IP_3_R-Grp75-VDAC1-MCU calcium regulation axis proteins mediated podocyte apoptosis, possibly by facilitating mitochondrial Ca^2+^ overload. In addition, antagonists to the axis could protect podocytes from ADR- and Ang II-induced apoptosis. Blocking the entry of Ca^2+^ into mitochondria could control proteinuria in the ADR nephropathy rat model. Further investigation into the pathway might facilitate the development of new drugs for podocyte-specific protection and proteinuria control.

## Abbreviations

ADR: Adriamycin
Ang II: Angiotensin II
Co-IP: Co-immunoprecipitation
Ctl: Control
ER: Endoplasmic reticulum
Grp75: Glucose-regulated protein 75
IP_3_: D-myo-inositol 1,4,5-triphosphate tripotassium salt
IP_3_R: Inositol 1,4,5-triphosphate receptor
MCU: Mitochondrial calcium uniporter
RR: Ruthenium red
VDAC1: Voltage-dependent anion channel 1
XeC: Xestospongin C
